# Primary care patients’ perspectives on the use of non-pharmacological home remedies in Geneva: a cross-sectional study

**DOI:** 10.1186/s12906-022-03564-7

**Published:** 2022-05-05

**Authors:** Neria E. Winkler, Paul Sebo, Dagmar M. Haller, Hubert Maisonneuve

**Affiliations:** 1 University Institute for Primary Care, Faculty of Medicine, University of Geneva, Geneva, Switzerland; 2 Department of Community, Primary Care and Emergency Medicine & Department of Paediatrics, Geneva University Hospitals, Geneva, Switzerland

**Keywords:** Home remedies, Non-pharmacological treatments, General practitioners, Primary care, Minor health problems, Views, Practices, Perspectives

## Abstract

**Background:**

Home remedies are anchored in patients’ everyday life, but their use in Western cultures remains scarcely explored. Our objectives were to investigate primary care patients’ perspectives and use of non-pharmacological home remedies in Geneva (Switzerland).

**Methods:**

In spring 2020, we conducted a cross-sectional survey among adult primary care patients in randomly selected general practices (*N* = 15). Patients were recruited in the waiting rooms and asked to complete a questionnaire about their sociodemographic characteristics, their home remedy use, and their expectations and reasons for using (or not using) home remedies. We employed descriptive statistics to summarise the data and logistic regression adjusted for clustering within practices to explore associations between home remedy use and participants’ sociodemographic characteristics.

**Results:**

Three hundred fourteen of three hundred ninety patients agreed to participate in the study (participation rate 80.5%). Home remedies were used by 64.4% of patients. The main reasons given were for preventive purposes (55.3%), self-care (41.0%), as an alternative to conventional medicine (40.5%) and to avoid or delay a medical consultation (38.5%). One-third of patients considered that it was the GP’s role to spontaneously inform them about home remedies (36.4%), another third considered that it was the GP’s role to inform them, but only upon specific request (32.3%), and the last third of patients declared that it was not the GP’s role to provide information about home remedies (30.3%). Patients living in an urban zone (adjusted OR 2.1; 95%CI 1.0–4.4; p 0.05) and those with a tertiary education background (adjusted OR 1.9; 95%CI 1.0–3.6; p 0.05) believed that it was their GP’s role to inform them about home remedies.

**Conclusions:**

Home remedies are used by a majority of primary care patients in Geneva. For a comprehensive and safe healthcare management in the context of patient-oriented medicine, more evidence-based research on efficacy and safety of home remedies as well as their place in primary care consultation is required.

## Background

Non-pharmacological treatments such as “home remedies” are ubiquitous in the media and non-scientific literature [1]. Despite their anchoring in patients’ everyday life [1–6] their use in Western cultures remains scarcely explored [1, 2, 4, 6].

In previous studies, patients declared that to treat common minor health problems they would like to be better informed by their general practitioner (GP) about alternatives to conventional medicine, and in particular non-pharmacological home remedies (NPHRs) [1, 6–8]. At the same time, most health professionals reported a lack of knowledge about alternative approaches to conventional medicine [9], and only a minority prescribed them in practice [6]. Resulting discrepancies could thus negatively impact the doctor-patient partnership [6]. Moreover, improper self-care practices are likely to entail detrimental consequences [10–12].

Improving GPs’ awareness and knowledge about the use of NPHRs, as well as the integration of self-care management education in this context, may potentially be a lever to improve the quality and safety of healthcare. This could lead to the appropriate integration of such self-care practices whilst strengthening the doctor-patient partnership [4, 6, 13–16].

To our knowledge, only a few studies in this field have been published in Europe until recently. In 2018, a survey among GPs explored the use of NPHRs in their daily practice [6]. Saline water, stretching exercises and applying cold were considered very useful NPHRs, even though they were not frequently prescribed in practice [6]. In the light of the growing interest in patient-centred medicine, a (more) balanced view of reliable information should benefit both GPs and their patients. We aimed, therefore, to complete the picture by exploring the patients’ perspectives on the use of NPHRs.

The objectives of this study were (i) to establish the prevalence of NPHR use among primary care patients, (ii) to explore patients’ expectations and reasons for using (or not using) NPHRs, and (iii) to identify associations between patients’ sociodemographic characteristics and NPHR use.

## Methods

### Setting

In 2020, this cross-sectional study was conducted by means of a self-administered questionnaire designed for adult primary care patients in the region of Geneva (Switzerland).

### Definition of NPHRs

Due to the absence of a unanimous definition of “home remedies”, the term is subject to various interpretations – not only by patients, but also by health professionals and researchers [1].

This may lead to an over- or underestimation of the prevalence of NPHR use. To avoid biases associated to misconception (e.g. study participants and researchers may be discussing different preparations), our research team agreed on the following working definition for the purpose of this study: NPHRs are remedies that “(i) cannot be obtained in a commercially available drug formulation and (ii) do not require external help from therapists” [6]. Consequently, medicinal products subject to prescription, over-the-counter drugs (OTCs) and herbal therapies (e.g. cranberry preparations, essential oils), as well as treatments provided by healthcare professionals (e.g. physiotherapy, osteopathy, hypnosis) and a large number of complementary and alternative medicine (CAM) methods (e.g. acupuncture, homeopathy, anthroposophical medicine, neural therapy) were excluded from our definition [6]. Hence, remedies for health in everyday life or in case of illness are considered; it may be plants or herbs, techniques, exercises or use of simple objects.

### Study site and study population

This study was designed as a cluster-randomised survey. The unit of randomisation was primary care providers (GPs), and the unit of analysis was patients. The data collection took place among adult primary care patients in the waiting rooms of randomly selected GP practices. The study population included patients 18 years of age or older, able to provide informed consent and to read and understand all study documents in French.

Patients presenting an acute emergency condition or who reported feeling too unwell to complete a study were excluded.

### Recruitment process

**Fig. 1:**
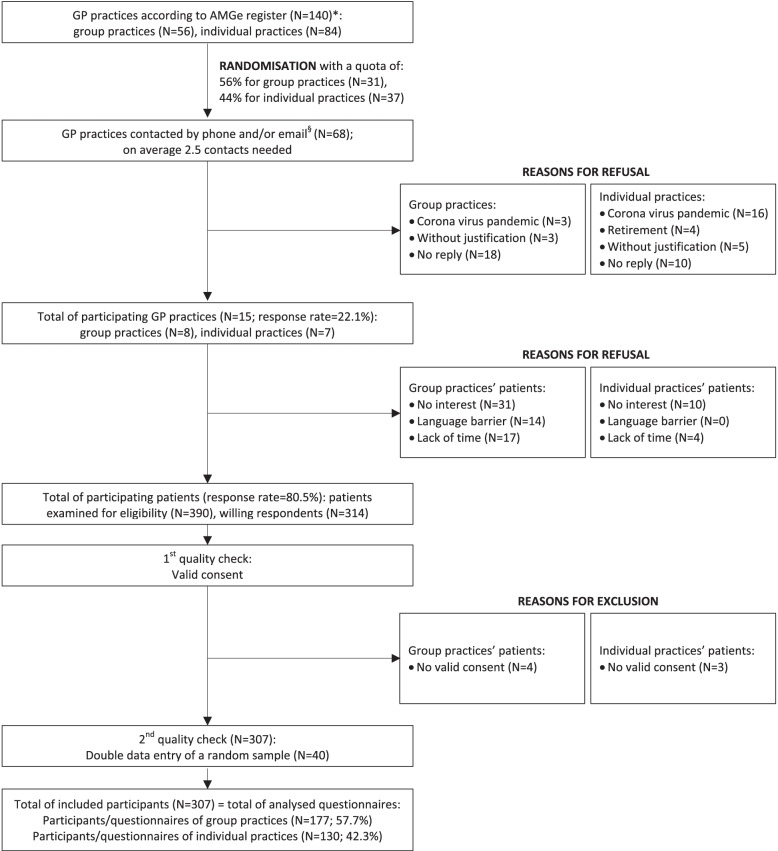
Recruitment of GP practices and patient flow. *Data are based on general information for Switzerland [17, 18], not specifically for Geneva. ^§^Three GP practices directly responded to first email contact (group practice *N* = 2; individual practice *N* = 1), four directly responded to first phone contact (group practice *N* = 3; individual practice *N* = 1), and eight had to be contacted again by phone and/or email (group practice *N* = 3; individual practice *N* = 5)

A random sample of GP practices in the region of Geneva was selected using the freely accessible online register of the association of physicians of Geneva (*Association des Médecins de Genève, AMGe*) (Fig. 1) [17]. The recruitment process was carried out by one of the study investigators (NEW). GP practices were invited to participate by email followed by up to three reminder phone calls per practice. Patients visiting their GP during consultation hours and independently of the study were consecutively included; participation in the study was voluntary. The co-investigator NEW was present in the respective waiting rooms of the consenting GPs and proposed the study to consecutive patients, informed them about the study conducted in French, and obtained written informed consent before distributing the self-administered questionnaire. NEW was available to answer any questions in an objective manner, without influencing patients’ answers.

### Measuring tool

Lack of validated questionnaires in this context required developing an original questionnaire based on the available literature and validated sociodemographic questionnaires [1, 19, 20]. We developed a self-administered questionnaire in French with a total of 16 questions consisting of an introductory key question followed by questions subdivided into three parts. We first assessed if the patients reported the use of NPHRs in the previous 12 months. Then, using hypotheses raised in a preliminary qualitative phase (unpublished observations; Ujupi D, Shabani V), we asked patients to rate eight statements summarising common reasons regarding the non-use of NPHRs (Table 1). We also included questions about patients’ sociodemographic characteristics using numeric rating scales (NRS), verbal rating scales (VRS) and open questions (Table 2). The following part of the questionnaire included variables generated from the aforementioned qualitative study (unpublished observations; Ujupi D, Shabani V), related to expectations and reasons for using NPHRs (Table 1). The last part of the questionnaire (not presented in this paper) explored patients’ specific use of a list of home remedies (*N* = 220) for common minor health problems (*N* = 58) (unpublished observations; Borsatti M), matching our working definition of NPHRs. Ongoing constructive discussions within our research group (*N* = 13) led to modifications of the questionnaire to improve its pertinence and accuracy. For feasibility reasons (i.e. length of the entire questionnaire), we decided to develop the questionnaire only in French, representing the official language of the study site, and to exclude patients who did not understand French.

**Table 1:** Prevalence of NPHR use (*N* = 307), participants’ views on NPHRs (*N* = 307) and GPs’ involvement in NPHR use (*N* = 195)

**Prevalence of NPHR use and participants’ views on NPHRs** (***N*** **= 307)**	**Number of participants (%) [95%CI]**
**Do you use NPHRs? (*N*** **= 306)**	
Yes	197 (64.4) [59.0–69.7]
No	109 (35.6) [30.3–41.0]
**For what reason(s) do you use NPHRs? (*N*** **= 195)**^a^	
For preventive purposes, to stay healthy or to avoid getting ill	108 (55.3) [48.4–62.4]
Because I can treat myself without the help of a therapist	80 (41.0) [34.1–47.9]
As an alternative to conventional medicine^b^	79^c^ (40.5) [33.6–47.4]
To limit the number of pharmacological treatments	53 (27.2) [20.9–33.4]
To avoid side effects associated with pharmacological treatments	43 (22.1) [16.2–27.9]
Because I do not trust pharmacological treatments	26 (13.3) [8.6–18.1]
Because I do not trust conventional medicine	15 (7.7) [4.0–11.4]
Because an effective pharmacological treatment does not exist	6 (3.1) [0.7–5.5]
To avoid or delay a medical consultation	75 (38.5) [31.6–45.3]
As a complement to pharmacological treatment	45 (23.1) [17.2–29.0]
When medical care seems too expensive for my health problem	18 (9.2) [5.2–13.3]
Other reasons^d^	7 (3.6) [1.0–6.2]
**For what reason(s) do you not use NPHRs? (*N*** **= 109)**^e^	
I do not know of any NPHRs	53 (48.6) [39.2–58.0]
I would rather see my GP than take NPHRs	42 (38.5) [29.4–47.7]
I have easy access to medical care and do not need to take NPHRs	39 (35.8) [26.8–44.8]
I prefer to use pharmacological treatments than NPHRs	17 (15.6) [8.8–22.4]
In my opinion NPHRs are ineffective	8 (7.3) [2.4–12.2]
Other reasons^f^	19 (17.4) [10.3–24.6]
**Do you think a GP’s role is to inform you about NPHRs? (*N*** **= 195)**^g^	**Number of participants (%) [95%CI]**
Yes	136 (69.7) [63.3–76.1]
Spontaneously on the initiative of my GP	71 (36.4) [29.7–43.2]
Only upon specific request from me	63 (32.3) [25.7–38.9]
No	59 (30.3) [23.8–36.7]
**Have you talked to your GP about your use of NPHRs? (*N*** **= 194)**^g^	
Yes^h^	65 (33.5) [26.9–40.2]
I brought it up spontaneously	52 (26.8) [20.6–33.0]
My doctor brought it up spontaneously	16 (8.2) [4.4–12.1]
Other reasons^j^	2 (1.0) [0.0–2.5]
No^i^	129 (66.5) [59.9–73.1]
I did not feel the need to talk to my GP about it	66 (34.2) [27.4–40.7]
My GP did not ask me about it	47 (24.2) [18.2–30.3]
I consider it a personal matter	23 (11.6) [7.3–16.4]
I forgot to tell my GP about it	10 (5.2) [2.0–8.3]
I consider that this practice is not part of medical care	8 (4.1) [1.3–6.9]
I fear my GP’s judgement of this practice	1 (0.5) [0.0–1.5]
Because I am afraid of being misunderstood by my GP	0 (0) [0.0–0.0]
My GP raised the subject, but I did not want to talk to him about it	0 (0) [0.0–0.0]
Other reasons^j^	7 (3.6) [1.0–6.2]

^a^Number of patients differs from 197 due to several possible responses

^b^Total differs from 100% due to several possible responses

^c^Number of patients differs from 79 due to several possible responses

^d^Other reasons (several possible answers): Because my GP didn’t prescribe pharmacological treatment (*N* = 3; 1.5%; 95%CI 0.0–3.2); Because I live or work in an area where it is difficult to consult a GP (*N* = 2; 1.0%; 95%CI 0.0–2.4); In combination with pharmacological treatment, as the maximum dose was reached (*N* = 2; 1.0%; 95%CI 0.0–2.4)

^e^Number of patients differs from 109 due to several possible responses

^f^Other reasons (several possible answers): My GP advised me not to use NPHRs (*N* = 1; 0.9%; 95%CI 0.0–2.7); I think NPHRs are too expensive (*N* = 1; 0.9%; 95%CI 0.0–2.7); Other (*N* = 17; 15.6%; 95%CI 8.8-22.4) not listed in detail due to the low representativeness

^g^Number of participants does not add up to 197 because of missing data

^h^Number of participants differs from 65 due to several possible responses

^i^Number of participants differs from 129 due to several possible responses

^j^Not listed in detail due to the low representativeness

**Table 2:** Participants’ sociodemographic characteristics (*N* = 307)

Characteristics (***N*** = 307)	Number of participants (%) [95%CI]	Mean (SD)
**Gender (*N*** **= 304)**		
Female	184 (60.5) [55.0–66.0]	
Male	120 (39.5) [34.0–45.0]	
**Age group (*N*** **= 305)**		52.1 (18.8)
< 40 years	86 (28.2) [23.2–33.3]	
40–59 years	110 (36.1) [30.7–41.5]	
≥ 60 years	109 (35.7) [30.4–41.1]	
**Place of residence (*N*** **= 307)**		
Urban zone	217 (70.7) [65.6–75.8]	
Semi-rural zone	65 (21.2) [16.6–25.7]	
Rural zone	25 (8.1) [5.1–11.2]	
**Nationality (*N*** **= 305)**^a^		
Swiss	217 (71.1) [66.1–76.2]	
French	40 (13.1) [9.3–16.9]	
Italian	19 (6.2) [3.5–8.9]	
Spanish	13 (4.3) [2.0–6.5]	
Portuguese	11 (3.6) [1.5–5.7]	
Other (< 2% per different nationality)	52 (17.0) [12.8–21.3]	
**Marital status (*N*** **= 301)**		
Married or living as a couple	160 (53.2) [47.5–58.8]	
Single	76 (25.2) [20.3–30.2]	
Divorced or separated	50 (16.6) [12.4–20.8]	
Widowed	15 (5.0) [2.5–7.4]	
**Family situation (*N*** **= 196)**		
With child/−ren	90 (45.9) [38.9–52.9]	
Without child/−ren	106 (54.1) [47.1–61.1]	
**Work status (*N*** **= 305)**		
Occupational activity	156 (51.2) [45.5–56.8]	
Retired	79 (25.9) [21.0–30.8]	
Student or apprenticeship/vocational training	26 (8.5) [5.4–11.7]	
Recipient of unemployment (ALV^b^) or invalidity (DI^b^) benefits^c^	22 (7.2) [4.3–10.1]	
Housewife/-husband	8 (2.6) [0.8–4.4]	
Other^d^ (mainly without employment)	14 (4.6) [2.2–6.9]	
**Completed training/education (*N*** **= 305)**		
University, FIT^e^, UAS^e^	120 (39.3) [33.9–44.8]	
Apprenticeship/vocational training	75 (24.6) [19.8–29.4]	
Baccalaureate or diploma from intermediate school	63 (20.7) [16.1–25.2]	
Compulsory schooling	42 (13.8) [9.9–17.6]	
No training/education^f^	5 (1.6) [0.2–3.1]	
**Self-estimated general health status (*N*** **= 304)**		
Excellent or very good	106 (34.9) [29.5–40.2]	
Good	150 (49.3) [43.7–55.0]	
Moderate or poor	48 (15.8) [11.7–19.9]	
**Number of daily medications (*N*** **= 295)**		2.0 (2.6)
0	103 (34.9) [29.5–40.4]	
1	58 (19.7) [15.1–24.2]	
2	46 (15.6) [11.5–19.7]	
≥ 3	88 (29.8) [24.6–35.1]	
**Number of consultations**^g^** to GP in the past 12 months (*N*** **= 306)**		
1	54 (17.6) [13.4–21.9]	
2–5	167 (54.6) [49.0–60.2]	
6–9	45 (14.7) [10.7–18.7]	
≥ 10	40 (13.1) [9.3–16.9]	
**Model of health insurance (compulsory health insurance) (*N*** **= 302)**		
Basic insurance with standard or optional deductible	191 (63.3) [57.8–68.7]	
General practitioner model	70 (23.2) [18.4–27.9]	
HMO (Health Maintenance Organisation) model	11 (3.6) [1.5–5.8]	
Telemedical model (Telmed or Callmed)	7 (2.3) [0.6–4.0]	
No-claims bonus	3 (1.0) [0.0–2.1]	
Other (mainly not knowing what kind of model)	20 (6.6) [3.8–9.4]	
**Annual deductible in Swiss Francs (*N*** **= 299)**		
300	141 (47.2) [41.5–52.8]	
500	62 (20.7) [16.1–25.3]	
1′000	11 (3.7) [1.6–5.8]	
1′500	13 (4.4) [2.0–6.7]	
2′000	4 (1.3) [0.0–2.6]	
2′500	26 (8.7) [5.5–11.9]	
Other^h^ (mainly not knowing the amount of annual deductible)	42 (14.0) [10.1–18.0]	

^a^Number of patients differs from 305 due to several possible responses (e.g. double citizen)

^b^*ALV* Unemployment Insurance, *DI* Disability Insurance

^c^Unemployment benefits (*N* = 4; 1.3%; 95%CI 0.0–2.6); invalidity benefits (*N* = 18; 5.9%; 95%CI 3.3–8.6)

^d^No unemployment benefits, no invalidity benefits

^e^*FIT* Federal Institute of Technology, *UAS* University of Applied Sciences

^f^Compulsory schooling not finished

^g^Only a personal meeting with the GP was defined as a consultation

^h^Not knowing the amount of annual deductible (*N* = 36; 12.0%; 95%CI 8.4–15.7); Preferring not to answer (*N* = 5; 1.7%; 95%CI 0.2–3.1); Insurance for WHO employees (*N* = 1; 0.3%; 95%CI 0.0–1.0)

### Statistical analyses and sample size determination

Data from paper questionnaires were digitised using the Qualtrics® research platform. The digitisation process was completed by a double data entry of 40 random samples to check the quality of the data entry. With an error rate of less than 5%, the quality of the digitisation process was considered adequate, and we did not perform a double data entry for the whole sample. We computed the prevalence of NPHR use by dividing the number of users by the total number of participating patients. We used frequency tables to describe categorical variables as well as means and standard deviations to summarise continuous variables. Associations between NPHR use and participants’ sociodemographic characteristics were explored by univariable and multivariable logistic regression and adjusted for clustering within GP practices. In the present paper, we did not analyse patients’ specific use of NPHRs collected in the last part of the questionnaire.

Calculation of statistical power was based on an estimated mean prevalence of NPHR use of about 75% [1, 4, 6, 7, 13, 21, 22]. We wanted a 95% confidence interval [95%CI] no wider than +/− 0.05. Given the formula for estimating a proportion and taking the clustering into account (intra-class correlation coefficient 0.05), the minimum required sample size was 288, anticipating 20 participants per practice. Taking into account missing data and difficulties in reaching patients, an additional 10% was targeted, resulting in recruitment of approximately 320 participants. Statistical significance was set at a two-sided *p*-value of ≤0.05. All analyses were carried out with Stata version 15.0. The data collected by means of the self-administered questionnaire are entirely presented in Tables 1 and 2, respectively, as formulated in the original questionnaire.

## Results

Of the GP practices approached, a total of 15 (participation rate 22.1%) agreed to have the study carried out in their waiting rooms, of which eight group practices (several practicing physicians; min 2, max 8) and seven individual practices (one single GP). Patients’ participation rate was 80.5% (*N* = 314). Our sample reflects the quota of patients consulting in individual (44%) and group practices (56%) in Switzerland [18, 22]. Figure 1 illustrates the study inclusion/exclusion process.

Table 2 presents participants’ main sociodemographic characteristics. Their median age was 52 years and the majority of participants were women (60.5%), Swiss (71.1%) and living in an urban zone (70.7%). According to the latest data published by the Federal Statistical Office (FSO), our sample represented Geneva’s multiculturalism with its foreign resident population (Geneva 2020: 39.8%) [22, 23].

### Prevalence of NPHR use and reasons for using (or not using) NPHRs

Nearly two-thirds (64.4%) of all participants reported using NPHRs (Table 1). They were mainly used for preventive purposes (55.3%), self-care (41.0%), as an alternative to conventional medicine (40.5%) (either to limit the number of medications taken (27.2%) or to avoid side effects associated with medications (21.1%)), and to avoid or delay a medical consultation (38.5%). By contrast, the main reasons for not using them were ignorance of NPHRs (48.6%), preference to consult their GP (38.5%) and easy access to medical care (35.8%).

### Patients’ expectations

About two-thirds of the users considered that it was the GP’s role to inform them about NPHRs, either spontaneously (36.4%) or upon specific request from patients (32.3%), whereas one-third thought that it was not his/her role (30.3%). Accordingly, two-thirds of the users did not talk to their GP about their use of NPHRs (66.5%) (Table 1).

### Univariable and multivariable analysis

Table 3 presents participants’ sociodemographic characteristics associated with NPHR use. While there was an initial discernible trend between NPHR use and female gender, this did not reach statistical significance in the multivariable analysis (adjusted OR 1.7; 95%CI 1.0–2.9, p 0.06). Table 4 shows participants’ sociodemographic characteristics associated with their expectations. Patients living in an urban zone and those with tertiary education background considered twice as strongly that it was their GP’s role to inform them about NPHRs (p 0.05). There were no other significant associations with patients’ sociodemographic characteristics.

**Table 3:** Associations between NPHR use and participants’ sociodemographic characteristics

Characteristics	Unadjusted OR (95%CI)	***p***-value*	Multivariate analysis	
			Adjusted OR (95%CI)	***p***-value^§^
**Gender**		0.04		0.06
Female	1.6 (1.0–2.6)		1.7 (1.0–2.9)	
Male	1		1	
**Age group**		0.28		0.92
< 40	1.5 (0.9–2.4)		1.3 (0.8–2.3)	
40–59	1.1 (0.7–1.8)		1.0 (0.6–1.7)	
≥ 60	1			
**Place of residence**		0.77		0.73
Urban zone	1.1 (0.7–1.6)		1.1 (0.7–1.7)	
Semi-rural or rural zone	1		1	
**Nationality**		0.06		0.22
Swiss	1		1	
Other	1.6 (1.0–2.5)		1.5 (0.8–2.7)	
**Completed training**		0.44		0.31
University, FIT^a^, UAS^a^	1.2 (0.7–2.0)		1.3 (0.8–2.0)	
Other	1		1	
**Self-estimated health status**		0.11		0.10
Excellent or very good	1		1	
Good	0.7 (0.4–1.1)		0.7 (0.5–1.1)	
Moderate or poor	1.1 (0.6–2.1)		1.3 (0.7–2.5)	

^a^*FIT* Federal Institute of Technology, *UAS* University of Applied Sciences

^*^Univariate logistic regression, adjusted for clustering within practices

^§^Multivariate logistic regression, adjusted for all variables listed in the table and for clustering within practice

**Table 4:** Associations between the view of GP’s role in informing about NPHRs and participants’ sociodemographic characteristics

Characteristics	Unadjusted OR (95%CI)	***p***-value*	Multivariate analysis	
			Adjusted OR (95%CI)	***p***-value^§^
**Gender**		0.04		0.07
Female	1		1	
Male	1.6 (1.0–2.6)		1.6 (1.0–2.5)	
**Age group**		0.51		0.81
< 40	1		1	
40–59	1.3 (0.7–2.1)		1.1 (0.6–2.1)	
≥ 60	1.2 (0.8–1.8)		0.9 (0.5–1.6)	
**Place of residence**		0.04		0.05
Urban zone	2.0 (1.0–3.8)		2.1 (1.0–4.4)	
Semi-rural or rural zone	1		1	
**Nationality**		0.28		0.12
Swiss	1.4 (0.7–2.7)		1.9 (0.9–4.3)	
Other	1		1	
**Completed training**		0.07		0.05
University, FIT^a^, UAS^a^	1.7 (1.0–3.1)		1.9 (1.0–3.6)	
Other	1		1	
**Self-estimated general health status**		0.82		0.86
Excellent or very good	1		1	
Good	1.2 (0.6–2.2)		1.2 (0.6–2.4)	
Moderate or poor	1.3 (0.5–3.2)		1.3 (0.5–3.7)	

^a^*FIT* Federal Institute of Technology, *UAS* University of Applied Sciences

^*^Univariate logistic regression, adjusted for clustering within practices

^§^Multivariate logistic regression, adjusted for all variables listed in the table and for clustering within practices

## Discussion

### Main findings

In this primary care study, the majority of participants reported regular use of home remedies, mainly for preventive purposes, self-care and as an alternative to conventional medicine. Only one-third of patients considered that it was the GP’s role to spontaneously inform them about home remedies, while another third considered that it was the GP’s role to inform them, but merely upon specific request. The last third of patients declared that it was not the GP’s role to provide information about home remedies.

### Comparison with existing literature

In accordance with existing literature, use of non-pharmacological treatments is highly prevalent among primary care patients [1, 4, 6, 7, 13, 21, 22]. In a German study conducted in 2013 in a primary care setting, Parisius et al. found that NPHRs were widely known and used by about 80% of participants [1]. Our results were consistent with the German study population, and similarly, NPHRs were essentially used for prevention, self-care and as an alternative to conventional medicine (i.e. for common minor health problems in primary care as stated by Finley et al. and Wändell et al.) [22, 24, 25].

Several studies underlined the association between female gender and NPHR use [1, 3, 4]. According to our results, there seemed to be an association between NPHR use and female gender, but this association did not reach statistical significance. Sebo’s study investigating GPs’ perspectives on NPHRs also showed an association between female gender and the prescription of NPHRs [6], suggesting that GPs employment of such remedies for their patients may reflect their private use. This is in line with the traditional conception that the knowledge of home remedies came from the grandmother and was passed on by the female descendants [4]. At that time when the conservative family model was even more pronounced, women took on the “care-giving role” by looking after and caring for the whole family [4, 6]. However, our findings suggest that female gender is associated with the view that it is not the GP’s role to address NPHR use. This might be due to their greater responsibility for their health, since women are more likely to be actively involved in healthcare decisions than men [4, 6].

As previously shown in other studies [1, 6, 7], there was little discussion about NPHRs between patients and GPs (*N* = 65; 33.5%). Contrary to other studies in which patients reported that they would like to be informed by their GP about NPHRs [1, 6, 7], two-thirds of participants in our study did not endorse this view. Surprisingly, only one-third of NPHR users considered that the GP’s role was to spontaneously inform them about NPHRs.

### Limitations

The canton of Geneva is a predominantly urban region. GPs participating in our study are not necessarily representative of all GPs practicing in Switzerland, as only 22.1% of the approached GPs agreed to participate, potentially representing a bias. However, the demographic profile of our patients is in line with the expected profile for our region. As all participants were recruited in the primary care context, our study does not necessarily reflect patients’ views in other healthcare contexts that may offer more common sources of information about home remedies.

In addition, comparisons with the few studies that have been published in this field are difficult, as there is no unanimous definition of home remedies. However, since this study is part of a research project on the use of NPHRs in primary care and paediatrics, currently performed by the University Institute for Primary Care of the Faculty of Medicine of Geneva, the same working definition was used for all studies. Consequently, comparison was easier with these studies, namely with Sebo’s study [6].

As to keep recall bias low, participants were asked about their NPHR use within the last 12 months. Recall bias should not be a problem per se since our project aimed at assessing only NPHRs used on a regular basis, and thus memory should not be impaired in this regard. During data collection it became apparent, however, that many patients were not always aware of their NPHR use, either because these were so naturally integrated into their habits or simply because they had not given thoughts to such use. Therefore, it might be assumed that the prevalence of NPHR use was somewhat underestimated in our study. The investigators refrained from further exploring this salience because it was considered a level of detail that could dilute the message of this study.

### Implications for research and practice

NPHR use is highly prevalent among primary care patients in Western countries, but only poorly integrated into the doctor-patient discourse. The idea that GPs should inform their patients more about NPHRs is being ubiquitously propagated [1, 6–8]. Our study sheds new light on this conclusion: according to two-thirds of NPHR users in our study, GPs are not expected to spontaneously inform them about NPHRs. These remedies are basically used for common minor health problems in primary care [22, 24, 25]. It therefore seems relevant for GPs to advise their patients only if there is a need, without systematically exploring the use of NPHRs of each patient.

## Conclusions

This is a pioneering study in Switzerland, giving an initial overview on the use of home remedies from adult primary care patients’ perspective. In the context of frequent common minor health problems, GPs may address NPHR use with caution, as it may conflict with the representations of the majority of their patients. Future studies should explore the representations of patients and GPs alike and the dynamics raised by the use of NPHRs in primary care.

## Data Availability

The datasets supporting the conclusions of this article are included within the article. The questionnaire designed and used for the purpose of the present research project is available in French from the corresponding author on reasonable request.

## Abbreviations

*NPHR/NPHRs*: Non-Pharmacological Home Remedy(ies)
*GP(s)*: General Practitioner(s)
OTCs: Over-The-Counter drugs
*CAM*: Complementary and Alternative Medicine
*NRS*: Numeric Rating Scales
*VRS*: Verbal Rating Scales
*AMGe*: Association des Médecins de Genève
*CCER*: Commission Cantonale d’Éthique de la Recherche
*FSO*: Federal Statistical Office
